# Spectral fingerprinting to evaluate effects of storage conditions on biomolecular structure of filter-dried saliva samples and recovered DNA

**DOI:** 10.1038/s41598-020-78306-1

**Published:** 2020-12-08

**Authors:** Raffaele Brogna, Harriëtte Oldenhof, Harald Sieme, Willem F. Wolkers

**Affiliations:** 1 Unit for Reproductive Medicine - Clinic for Horses, University of Veterinary Medicine Hannover, Hannover, Germany; 2 Biostabilization Laboratory - Lower Saxony Centre for Biomedical Engineering, Implant Research and Development, University of Veterinary Medicine Hannover, Stadtfelddamm 34, Hannover, 30625 Germany

**Keywords:** Infrared spectroscopy, DNA, Biochemical assays, Diagnostic markers, Assay systems

## Abstract

Saliva has been widely recognized as a non-invasive, painless and easy-to-collect bodily fluid, which contains biomarkers that can be used for diagnosis of both oral and systemic diseases. Under ambient conditions, salivary biomarkers are subject to degradation. Therefore, in order to minimize degradation during transport and storage, saliva specimens need to be stabilized. The aim of this study was to investigate the feasibility of preserving saliva samples by drying to provide a shelf-stable source of DNA. Human saliva was dried on filters under ambient conditions using sucrose as lyoprotective agent. Samples were stored under different conditions, i.e. varying relative humidity (RH) and temperature. In addition to assessment of different cell types in saliva and their DNA contents, Fourier transform infrared spectroscopy (FTIR) was used to evaluate the effects of storage on biomolecular structure characteristics of saliva. FTIR analysis showed that saliva dried without a lyoprotectant exhibits a higher content of extended β-sheet protein secondary structures compared to samples that were dried with sucrose. In order to evaluate differences in characteristic bands arising from the DNA backbone among differently stored samples, principal component analysis (PCA) was performed, allowing a clear discrimination between groups with/without sucrose as well as storage durations and conditions. Our results indicated that saliva dried on filters in the presence of sucrose exhibits higher biomolecular stability during storage.

## Introduction

Human saliva contains a variety of compounds that are associated with pathophysiological conditions. Presence and/or quantitative changes of salivary biomarkers can be used for diagnosis of both oral and systemic diseases^1,2^. Due to the highly permeable and rich vasculature surrounded the salivary gland, there is free exchange between blood and adjacent saliva-producing acinus cells^3,4^. Therefore, it has been suggested that saliva may serve as alternative to blood-based diagnostic. From saliva, however, lower immunoglobulin and DNA amounts can be recovered as compared to blood^5,6^. Nonetheless, saliva has been widely recognized as a non-invasive, painless and easy-to-collect bodily fluid. In addition, saliva requires less manipulation than blood, it does not clot, and it appears safer for handling and associated infective transmission risks^3,7^. Saliva based-assays for various uses are exponentially growing, and range from pregnancy testing to alcohol and drugs detection^8^. Furthermore, numerous studies exist on the use of saliva protein markers for the diagnosis of systemic cancers^9^. Levels of vascular endothelial growth factor (VEGF), epidermal growth factor (EGF) and carcinoembryonic antigen (CEA), for example, have been described to be significantly elevated in breast cancer patients^10^. In addition to protein/antigen based tests (e.g. ELISA: enzyme-linked immunosorbent assay), also nucleic acid based (e.g. PCR: polymerase chain reaction) methodologies are applicable on saliva samples. The latter has been used for early detection of infective diseases, including *Ebola*, *Herpes simplex*, *Epstein-Barr* and *Cytomegalo* viruses^3^.

Under ambient conditions, protein and nucleic acid based biomarkers in bodily fluids are subject to degradation. In order to minimize degradation during prolonged storage, e.g. in a biobank, specimens need to be stabilized. This can be done by lowering the temperature down to refrigeration temperatures (i.e. 4 °C) or down to (ultra)-low subzero temperatures (i.e. ‒80 °C or in liquid nitrogen). Preservation of saliva in a stable dry state would simplify bioanalytical applications. More specifically, this would allow for storage and transport at ambient temperatures. With dry storage, there is no need for liquid nitrogen tanks and/or freezers, which reduces the carbon footprint associated with cryogenic storage. Moreover, storage of dry specimens can be implemented in non-laboratory settings and in underdeveloped countries or regions. Spot drying of saliva combined with mass spectrometry has been successfully used in drug content analysis^11^, as well as detection of specific bacteria^12^.

Bodily fluids are primarily composed of water (~ 88%). Upon drying, biomolecules may undergo conformational changes due to the loss of hydrogen bonding with water and due to temperature changes during the drying process. During air-drying, the sample temperature drops due to evaporative cooling, which is due to the kinetic energy loss associated with transforming liquid into gas. Biomolecular damage can be reduced when drying is done in the presence of non-reducing sugars. Disaccharides like sucrose protect biomolecules by replacing hydrogen bonds normally existing with water^13,14^. In addition, they facilitate the formation of a highly viscous glassy state in which structures are embedded and chemical reactions are slowed down^15^. Stability in the glassy state is affected by the storage conditions, particularly the relative humidity, which affects the glass transition temperature (Tg). If the storage temperature is too close to Tg, molecular mobility increases and physicochemical deteriorative reactions take place^16,17^.

The aim of this study was to evaluate the feasibility of air-drying on polyvinylidene fluoride (PVDF) circular membrane filters to preserve human saliva samples for use in diagnostic applications in cohort studies. First, cellular and DNA recovery from saliva samples obtained using different collection methods (‘swab’ and ‘spitting’) were investigated. Different drying approaches were studied and the beneficial effects of sucrose as lyoprotective agent was tested. Fourier transform infrared (FTIR) spectroscopy was used to evaluate storage stability of dried saliva samples by investigating changes in biomolecular structures using principal component analysis (PCA) of different spectral regions.

## Results

### Cell concentration, composition, membrane intactness and DNA recovered from human saliva collected using different approaches

**Figure 1:**
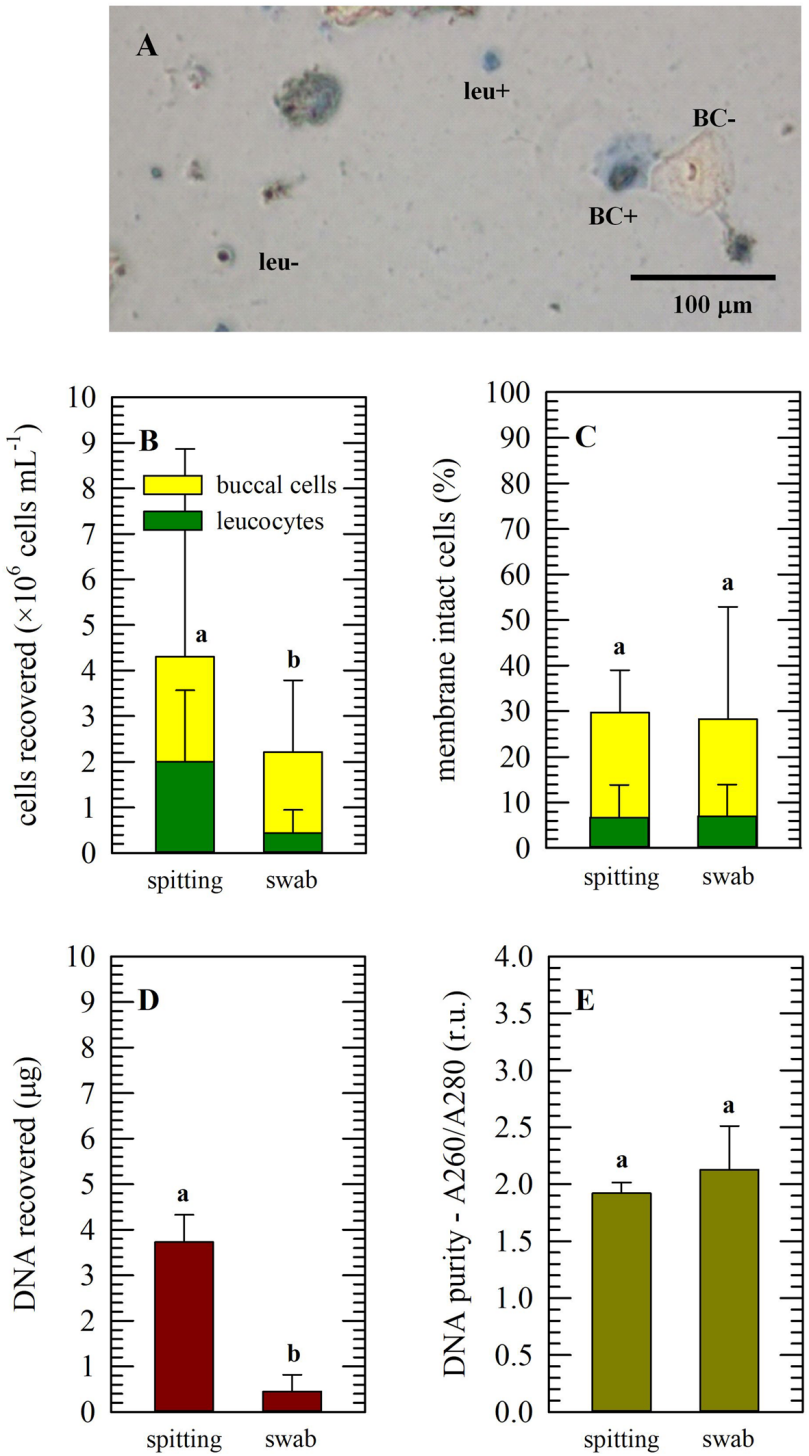
Cell concentration, composition and membrane intactness of saliva samples, as well as DNA contents that were recovered. Saliva (100 µL) was collected via spitting directly in a tube, or via using a buccal swab (2 × 6 × scrubbing) for transfer into saline (100 µL). Microscopic observations of trypan blue stained specimens (**A**) show presence of leukocytes (leu) and buccal cells (BC), whereas membrane damaged cells exhibit intracellular blue staining ( +). Cell concentrations were determined using a hemocytometer (**B**), while also discriminating between numbers of membrane intact/damaged cells (**C**) and cell types (buccal cells: yellow, leucocytes: green). In addition, total DNA was extracted and its content was determined, with respect to the original saliva/saline volumes recovered indicated above (**D**). DNA purity was evaluated by determining the absorbance values at 260 versus 280 nm (**E**). Mean ± standard deviations are presented, originating from saliva samples from twenty different donors, and different letters represent significant differences between collection methods (*p* ≤ 0.05) as determined using a student's t-test.

Saliva samples from different donors were collected via spitting or using swabs, where after specimens were analyzed microscopically (Fig. 1A–C). Figure 1A depicts a typical micrograph, after staining with trypan blue to discriminate between membrane intact and damaged cells, and different cell types. It should be noted that a typical procedure was followed with a swab (i.e. scrubbing each cheek 6 ×); however, recovered cell numbers presumably can be increased by increasing the scrubbing. With spitting, large variations amongst donors were found, but on average about equal amounts of buccal cells and leucocytes were recovered. The relative number of leucocytes obtained with swabs was lower (Fig. 1B). Irrespective of the method applied, the percentage of membrane intact cells was only about 30%, of which ~ 5% were leucocytes (Fig. 1C). In agreement with the different cell numbers that were collected, higher DNA amounts were recovered for spitting as compared to using swabs; namely 3.7 ± 0.60 µg from 100 μL of saliva versus 0.44 ± 0.36 µg from buccal swabs (Fig. 1D). No apparent differences in A260/A280 values indicative for DNA purity were found (Fig. 1E). Saliva collected via ordinary spitting in tubes was found most convenient, and therefore used for further studies.

### Drying kinetics of saliva on different surfaces and exposure to different drying conditions

**Figure 2:**
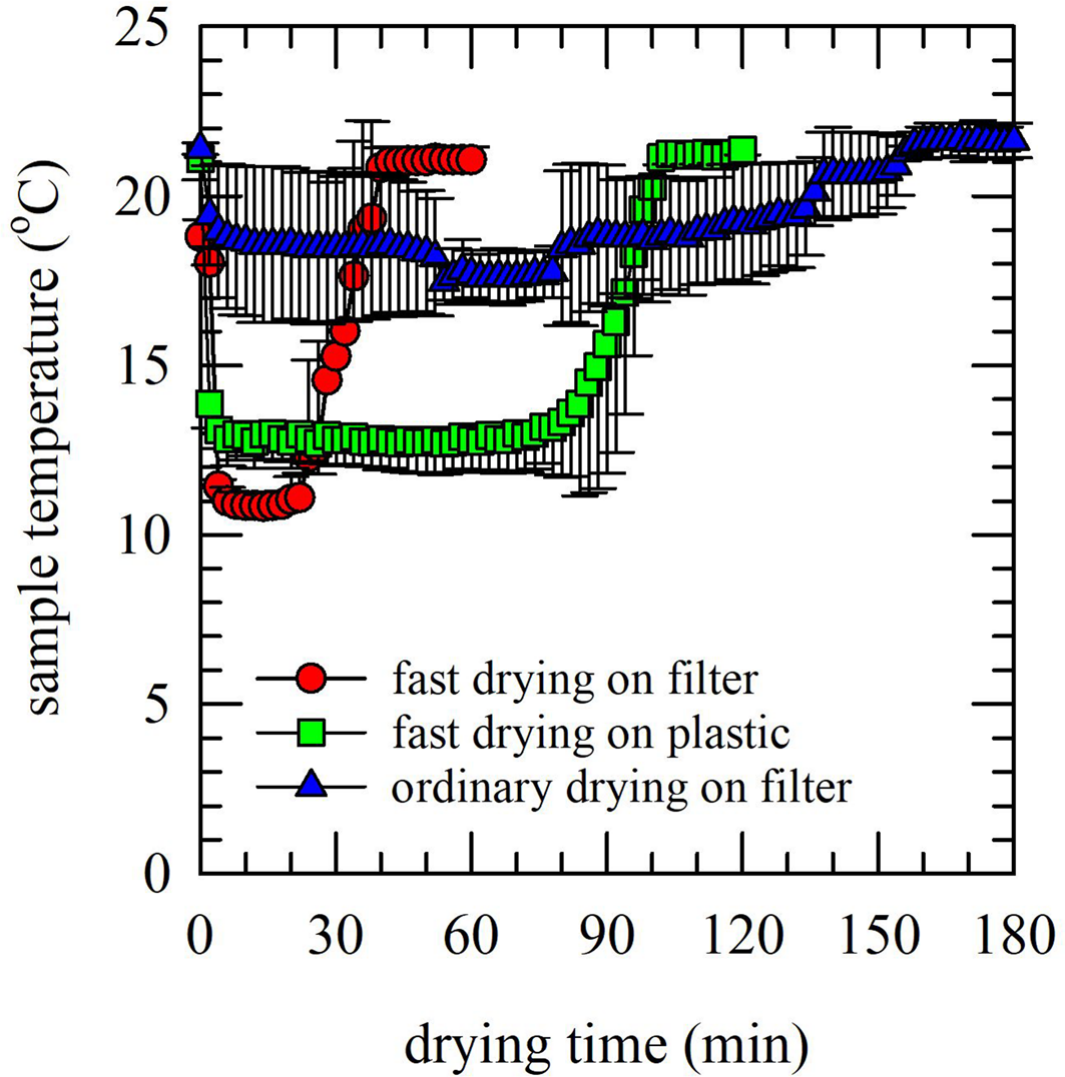
Drying kinetics of saliva samples deposited on different surfaces and exposed to different drying conditions, as determined by following the sample temperature versus the drying time. Saliva was dried fast under a stream of dry air, on filters (red circles) or on a plastic surface (green squares). In addition, filters with saliva were exposed to ordinary air drying (blue triangles). Pooled saliva was used from three different donors. Measurements were performed in triplicate using saliva aliquots, and mean values ± standard deviations are presented.

Drying kinetics was investigated for saliva samples deposited on different surfaces. Special emphasis was placed on the time needed for obtaining a dry state, and associated temperature changes. Figure 2 shows that drying and water evaporation coincides with a decrease in the sample temperature, followed by a return to the original temperature when attaining the dried state. This phenomenon is explained by removal of latent heat (i.e. energy release) from the evaporation surface. The kinetics are dependent on the drying conditions, with faster drying resulting in reaching a lower minimum temperature (i.e. down to 10 − 13 °C). Drying of saliva was fastest on PVDF filters using a stream of dry air (~ 40 min; for 1 mL), whereas drying on a plastic surface using dry air took much longer (~ 100 min). With ordinary drying of saliva on filters, without applying a stream of dry air, the drying time increased to ~ 180 min. In the absence of dry air, the decrease in sample temperature due to evaporation was much less (i.e. stayed above ~ 17 °C compared to ~ 12 °C in the presence of dry air). No differences in drying kinetics were seen for specimens dried with or without 10% sucrose (data not shown). It was assumed that faster drying is beneficial, therefore saliva was dried on PVDF filters in the presence of a stream of dry air to accelerate the drying process.

### Sub-optimal storage of saliva dried on filters for DNA extraction

**Figure 3:**
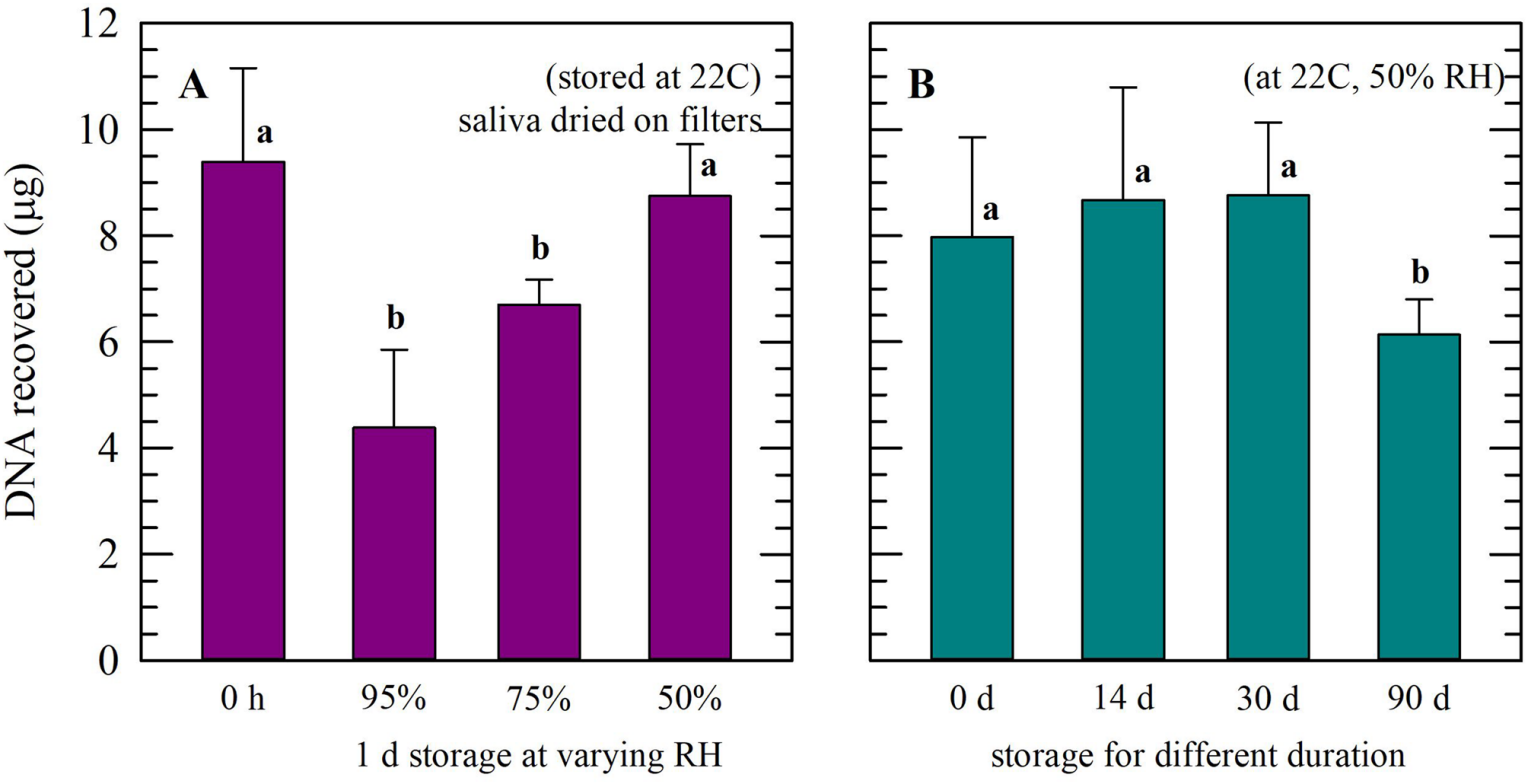
DNA recovery of saliva samples (250 µL), after drying on filters and storage under different conditions. Recovered DNA contents were determined before and after 1 d storage at different relative humidity conditions (**A**; 50%, 75%, and 95% RH), as well as after storage under ambient conditions (22 °C, 50% RH) for different durations (**B**; 0, 14, 30, and 90 d). Pooled saliva was used from three different donors. Measurements were performed in triplicate, and mean values ± standard deviations are presented. Different letters represent significant differences between storage conditions/storage times (*p* ≤ 0.05) as determined using a one-way ANOVA test.

To assess storage stability of dried saliva samples and feasibility to use for DNA recovery, total DNA was extracted from saliva dried on filters after storage, under different (i.e. sub-optimal) conditions. Here, 1 mL saliva was added per filter and, for storage, filters were divided in four equal parts containing approximately 250 µL saliva each. First, dried specimens were stored for 1 d at increasing relative humidity (Fig. 3A). It was found that 1 d storage at ambient relative humidity (~ 50% RH) did not affect the DNA amount that could be recovered, and similar amounts were determined compared to specimens not subjected to dried storage (8.75 ± 0.97 versus 9.36 ± 1.87 µg; from 250 µL saliva). With exposure to high relative humidity conditions, however, the amount of DNA recovered after storage clearly decreased, (4.38 ± 1.47 and 6.70 ± 0.48 µg for 95 and 75% RH, respectively). Even though DNA amounts recovered after dried storage under ambient conditions (~ 22 °C, ~ 50% RH) without protective measures remained unaffected after longer storage durations of 90 d, lower recovered DNA contents were found after longer storage duration (Fig. 3B), indicating degradation took place. In samples supplemented with 10% sucrose, a similar trend was observed (data not shown).

### FT-IR spectral analysis of biomolecular structures in saliva samples dried and stored on filters

**Figure 4:**
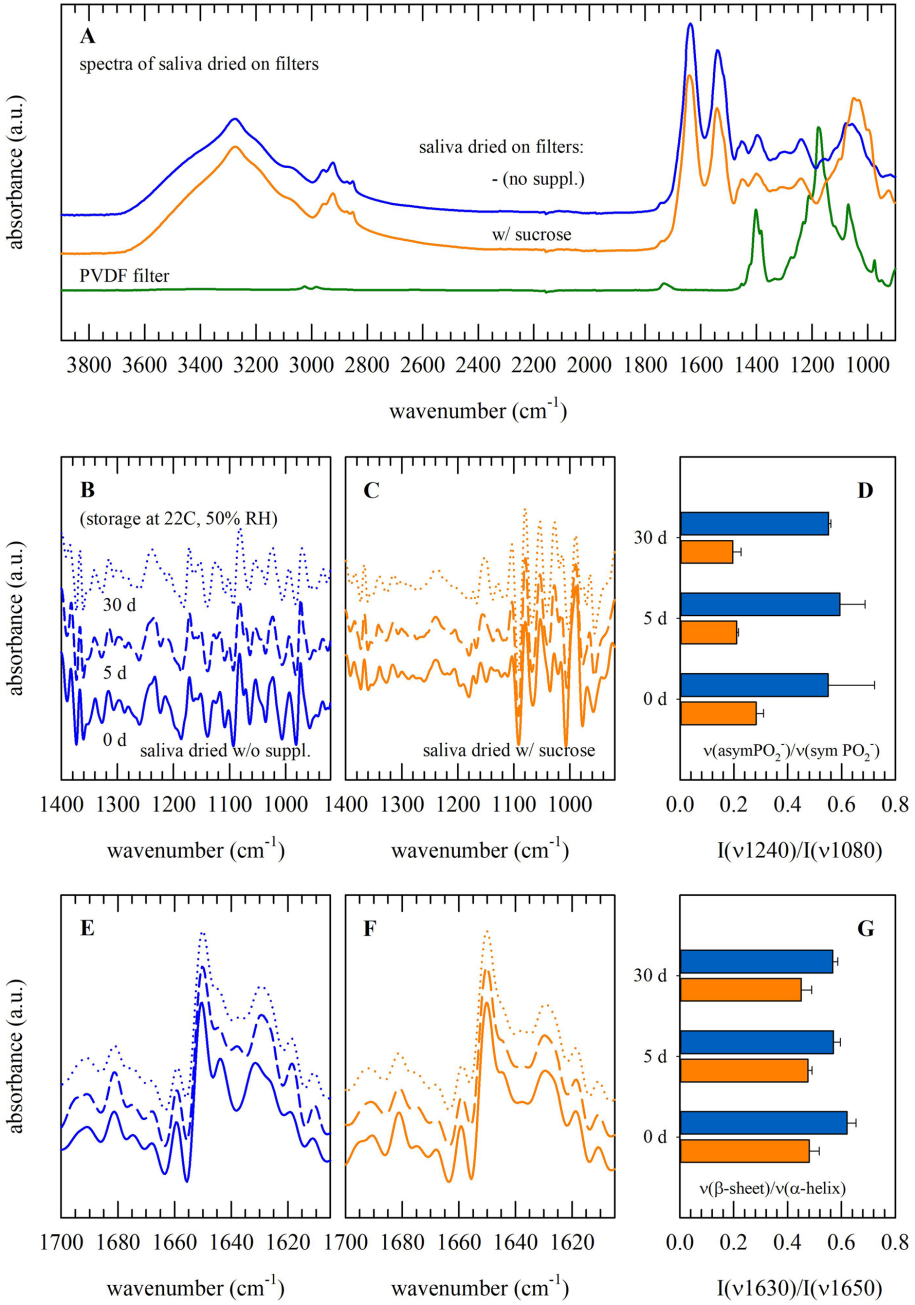
FT-IR spectral analysis of saliva dried on filters. Spectra were collected for PVDF filters only (green lines), as well as filters with saliva dried without protectants (blue lines and bars) or dried with 10% sucrose (orange lines and bars). Spectra were acquired directly after drying (**A**; 0 d) as well as during storage at ambient conditions (**B**–**G**; 0 d, 5 d and 30 d). Full original spectra are presented (**A**) as well as normalized second derivative spectra of the fingerprint (**B**, **C**) and amide-I (**E**, **F**) regions. For depicting possible differences amongst samples, specific absorbance band intensity ratios were calculated. These included ratios for the asymmetric versus symmetric PO2- stretching vibration bands [D; I(v1240)/I(v1080)], and the β-sheet versus α-helical structure bands [G; I(v1630)/I(v1650)]. Pooled saliva was used from three different donors. Measurements were performed in triplicate, and mean values ± standard deviations are presented.

In order to obtain more insights in biomolecular structure and stability, saliva dried on filters was evaluated using FT-IR. Figure 4A shows full original spectra of saliva dried on filters with or without sucrose. In addition, PVDF/filter-specific peaks were resolved. CH-stretching vibrations, which originate from lipids and proteins, are found in the 3000 − 2800 cm^−1^ region. CO-stretching and NH-bending vibrations from the protein backbone give rise to respectively the amide-I (~ 1640 cm^−1^) and -II (~ 1550 cm^−1^) absorbance bands. In the so-called fingerprint region, below 1500 cm^−1^, a variety of characteristic infrared bands can be observed that are difficult to assign to specific molecular group vibrations. This region includes characteristic sugar and DNA regions at respectively, 1100–700 cm^−1^ and 1400 − 960 cm^−1^. Formation of a (protective) glassy state by sugars is typically evident from the shape and broadening of the OH-stretching vibration band (3600–3000 cm^−1^).

For specimens subjected to storage, first, absorbance bands arising from DNA were inspected. In order to resolve different bands more clearly, normalized second derivative spectra were generated and compared (Fig. 4B,C). For quantification, the band intensities of the asymmetric and symmetric stretching PO_2_^−^ vibration were determined and their ratio was calculated. This revealed higher I(*v*1240)/I(*v*1080) values for saliva samples dried and stored without sucrose, whereas no differences in this ratio were observed during ambient storage for up to 30 d (Fig. 4D).

To reveal possible differences in the overall protein secondary structure amongst samples, the amide-I (1700‒1600 cm^−1^) region was inspected (Fig. 4E,F). From this analysis, it was evident that the relative content of extended β-sheet structures was higher for saliva dried on filters without protectant/supplements, as compared to samples dried with sucrose. This is expressed as a lower value for the band intensity ratio β-sheet versus α-helical structures, [I(*v*1630)/I(*v*1650]; and implies that adding sucrose prevents dehydration-induced changes in protein secondary structure (i.e. denaturation). No further changes in the overall protein secondary structure were observed during storage for up to 30 d (Fig. 4G).

**Figure 5:**
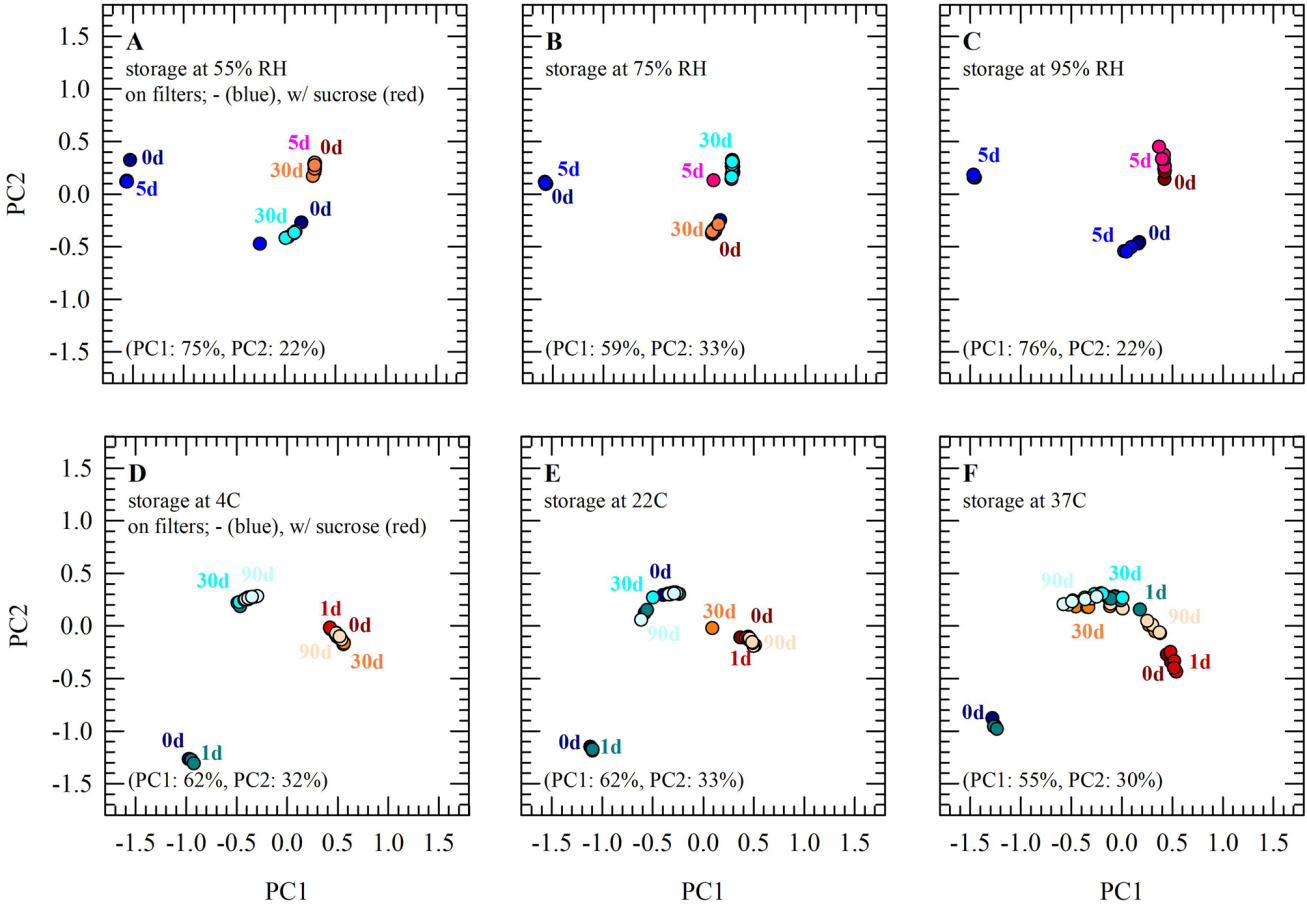
Principal component analysis of spectra obtained for saliva dried with(out) sucrose on filters during storage. PCA was performed for the fingerprint region (1400 − 900 cm^−1^), after vector normalization of the spectra. Saliva was dried without supplements (blue symbols) or with 10% sucrose (red/orange symbols), and stored for up to 90 d (lighter colors for later time points). Specimens were stored at 22 °C under different relative humidity (**A**: 50% RH, **B**: 75% RH, **C**: 95% RH). Furthermore, specimens were sealed under ambient conditions and stored at different temperatures (**D**: 4 °C, **E**: 22 °C, **F**: 37 °C). Pooled saliva was used from three different donors. All measurements were performed using six sample aliquots per treatment group.

In addition to inspecting changes in specific spectral absorbance bands of saliva dried and stored under different/sub-optimal conditions, PCA was applied on the fingerprint region (Fig. 5). In general, it can be said that PC1 describes most of the observed variance and allows discrimination between specimens with or without sucrose and different storage conditions. PC1 versus PC2 plots show good separation between specimens dried without and with protectants, which likely can be attributed to the presence or absence of sucrose bands. Furthermore, PC1 versus PC2 plots show that initial data points for specimens dried and stored without sucrose are clearly separated from each other, whereas this is not the case for specimens dried with sucrose. In case when samples were stored at RHs lower than 75%, clusters with data points appear more compact for specimens supplemented with sucrose (Fig. 5A–C). Moreover, cluster size/compactness was dependent on the storage temperature (Fig. 5D–F). Especially in case when samples were stored at 37 °C (i.e. exposure to accelerating aging), cluster sizes appeared to increase, both for specimens with or without sucrose. An increase in the data cluster size may be indicative of increased sample heterogeneity due to chemical and/or conformational changes during storage. Saliva dried on filters with sucrose and stored for up to 30/90 d at 22 °C and 75% RH exhibited only minor spectral changes.

### FT-IR spectral analysis of DNA extracted from saliva dried and stored on filters

**Figure 6:**
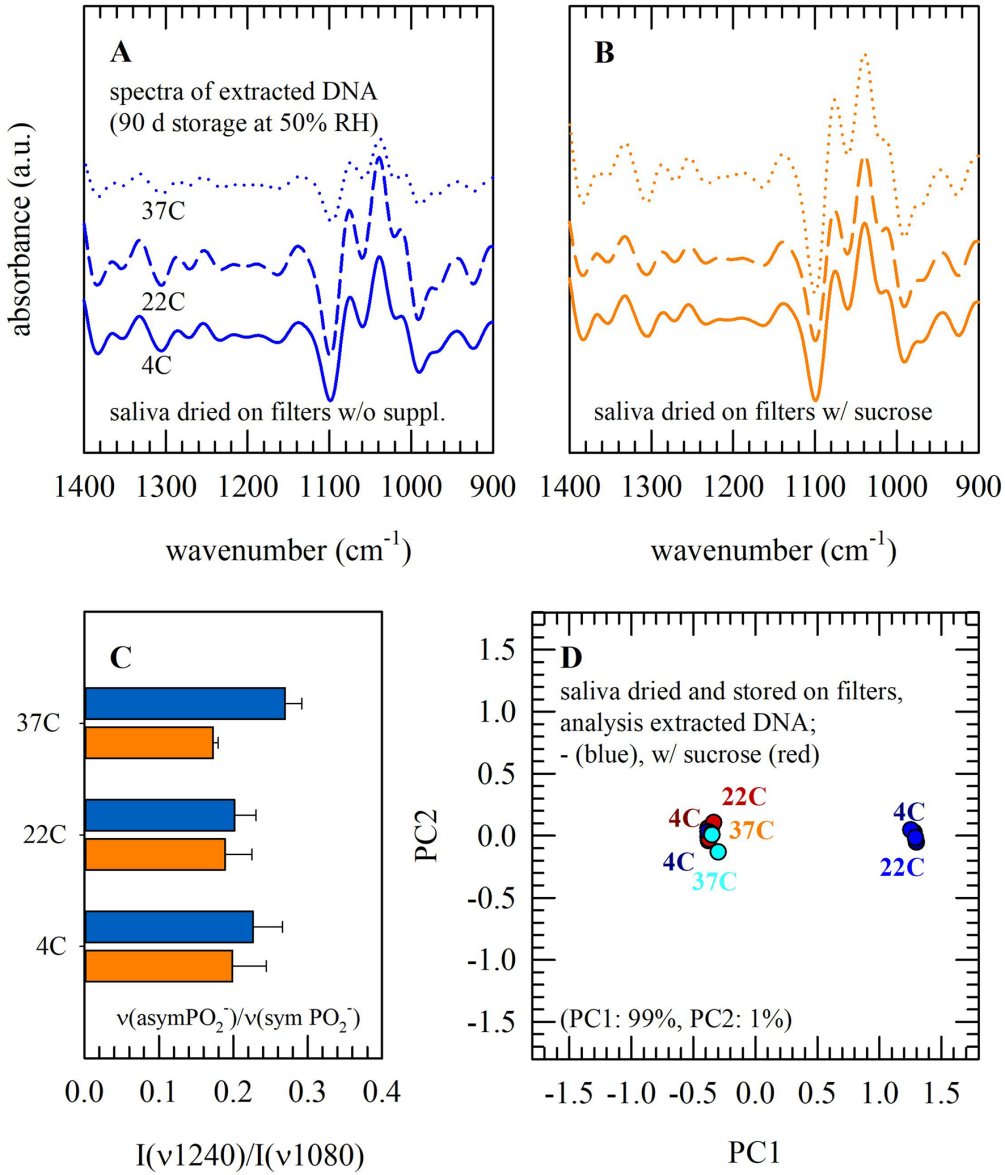
FT-IR spectral analysis of DNA extracted/recovered from saliva dried and stored on filters. Saliva was dried on filters without supplements (blue lines, bars and symbols) or with 10% sucrose (orange/red lines, bars and symbols), and stored for 90 d at different temperatures (4, 22, and 37 °C). Normalized second derivative spectra of the fingerprint spectral region are presented (**A**,**B**), as well as a comparison of the band intensity ratios of the asymmetric versus symmetric PO2- stretching vibration [**C**; I(v1240)/I(v1080)]. Furthermore, the 1400 − 900 cm^−1^ spectral region was subjected to PCA and score plots of the first two principal components were prepared (**D**; with lighter colors for higher temperatures). Pooled saliva was used from three different donors. Measurements were performed in triplicate, and mean values ± standard deviations are presented.

In addition to inspecting the overall biomolecular structure of saliva dried on filters, DNA was extracted from the filters and subjected to spectral analysis (Fig. 6). This was done for specimens with(out) sucrose stored at 4 − 37 °C, for 90 d. Figure 6A,B shows infrared absorbance spectra of recovered DNA, after drying on the ATR crystal. In this case, there are no spectral contributions of sucrose since it is (presumably) removed during the extraction procedure. In addition to the asymmetric and symmetric stretching PO_2_^−^ vibrations, dried DNA exhibits prominent absorbance bands at ~ 1050 and ~ 965 cm^−1^ (i.e. C-O and C–C stretching vibrations of the DNA backbone). When specimens were stored at 37 °C, in the absence of sucrose, the intensities of the DNA-backbone C-O and the symmetric PO_2_^−^ stretching vibrations both decrease_._ This is assumed to be associated with DNA structural damage (i.e. loss of structural integrity). Also, I(*v*1240)/I(*v*1080) values are higher for DNA recovered from saliva dried on filters without sucrose (Fig. 6C). PCA of extracted DNA spectra indicates that, when saliva was dried and stored with sucrose, minor changes occur during storage at temperatures ranging from 4 − 37 °C (Fig. 6D). In contrast, in the absence of protective supplements, spectra of DNA extracted from specimens stored at 37 °C appear different and form a separate cluster from those of 4 − 22 °C.

## Discussion

The advantage of saliva compared to other fluids such as blood, serum, or plasma is that it is non-invasive for volunteers. DNA and other biomolecules in saliva are subject to degradation when stored at ambient conditions, and therefore saliva samples have to be processed and stabilized within hours so that the samples can be safely stored for analysis at a later time point. It is shown here that saliva samples can be dried within two hours when samples are dried on a filter in the presence of dry air. In the absence of a dry purge air system, drying takes considerably longer even on filters, but in this case smaller sample volumes can be used, or the sample could be spread over a greater surface on a filter. In this study, we have evaluated storage stability of filter-dried saliva samples under ambient as well as sub-optimal conditions. Two collection methods were tested and it was found that spitting allowed for a more easy recovery of cells as compared to cell collection using a swab. Saliva samples consist of different cell types. Factors that have an impact on the cell composition in the oral cavity are not completely understood, but health status is one of them. Depending on age and health, human saliva is composed of ~ 75% epithelial buccal cells and ~ 25% leukocytes^19,20^. Our findings here are in agreement with these findings. Salivary leukocyte contents are typically higher in patients with oral cavity inflammation or gingivitis^21,22^. Saliva samples obtained from swabs are enriched with squamous epithelial buccal cells when compared with samples obtained by spitting^20^. Leukocytes have been identified as an excellent source for obtaining large quantities of DNA^23^, however, due to their ectodermal origin, buccal epithelial cells are considered a better comparison to peripheral blood^24^. The latter is especially relevant for epidemiological studies and studies on epigenetic characteristics^25^.

Exposure of biological specimens to drying results in drastic changes in their physical–chemical properties^13,26^. Changes in molecular interactions occurring during the removal of water bound, lead to biomolecular phase and structural changes, such as protein unfolding^27^ and lipid fluid-to-gel phase changes^28^. Here, FTIR analysis showed that saliva dried without a lyoprotectant exhibits a higher content of extended β-sheet protein secondary structures compared to samples that were dried with sucrose. In addition, reactive oxygen species (ROS) levels such as superoxide anion radicals increase, which in turn may react with biomolecules (i.e. lipids, proteins, nucleic acids) therewith impairing their function^29^. Damage due to ROS accumulates during storage in dried state resulting in degradation of biomarkers of interest, e.g. DNA in cells^30^. Oxidative damage results in DNA strand breakage and consequently decreases of DNA supercoil content and transfection efficiency^31^. Principal component analysis conducted on FTIR spectra allowed a clear discrimination between groups with/without sucrose, and storage experiments indicated that saliva dried on filters in the presence of sucrose exhibits higher biomolecular stability during storage. When packaged in chromatin, DNA is more stable than naked DNA; however, due to the presence of oxidizing compounds (e.g. peroxyl radicals), purified DNA is more stable than DNA in cells or tissues^32^. Oxidative damage may explain the observed decrease in DNA recovery when samples are stored at high relative humidity.

Different processing methods can be used for removal of water from biospecimens. These include convective, vacuum and freeze-drying^33,34^. Freeze-drying is widely used in pharmaceutical and food applications, because the drying process is robust, reproducible, and can easily be scaled up^35^. Vacuum, foam and spray drying can also be used for larger samples and are typically employed for processing and stabilization of proteins, enzymes and vaccines^33,36–38^. Convective drying is more difficult to control. Drying saliva on filters has been applied for easy sampling, handling and extraction of lidocaine^11^.

The drying rate during convective drying is dependent on the temperature, the relative humidity, and the surface on which the sample is positioned. A faster drying rate is considered beneficial since it shortens the duration in the less stable hydrated state during which biomolecules are subject to degradation. The porous structure of filters shortens the drying, whereas applying a stream of dry air further shortens the drying time^39^. In our study, drying of saliva on filters while applying a stream of air resulted in faster drying and allowed for recovery of large quantities of DNA. However, if dry air is not available, e.g. when samples need to be collected in non-laboratory settings, natural drying on filters is a good alternative. In this case, the relative humidity surrounding the sample should be kept low, e.g. by using drierite desiccant bags. DNA extracted from saliva has been successfully used in studies involving microarray genotyping and next generation sequencing^40,41^. For this purpose, saliva samples were stored at room temperature when used in short-term or for longer durations at 4 °C^41^. It has been described, however, that storage per se, temperature and relative humidity conditions provoke DNA damage and affect, for example, methylation patterns^42^. Sugars need to be added as lyoprotective agents to minimize biomolecular damage and to ensure glass formation. The lyoprotective properties of disaccharides, both in nature as well as in pharmaceutical applications, are well established^27,28,43^.

FT-IR studies suggested that biomolecules in saliva specimens appear more stable when supplemented with sucrose prior to drying, i.e. in case when saliva was dried on filters and stored for up to 3 months at 22 °C and 75% RH or less severe conditions. This was evident in PCA score plots as subsequent data points appearing in close proximity versus data points more distant from each other.

Specific infrared absorbance bands have been assigned to different DNA conformational structures, associated with B, A and Z-like transitions and base-specific interactions^44,45^. Band assignments of spectra from cellular samples, however, are more complex as those of isolated biomolecules^46^ since different structures and interactions may be adopted dependent on the microenvironment^46,47^. In infrared spectra of both saliva on filters and extracted DNA samples, prominent peaks where observed at ~ 1240 and ~ 1090 cm^−1^, which likely can be attributed to DNA-specific antisymmetric and symmetric PO_2_^−^ stretching vibrations^45,48,49^. For isolated DNA, the asymmetric/symmetric phosphate band ratio has been used to discriminate among different DNA compositions and relative double- versus single-stranded contents^48^. In addition, we found a temperature-dependent alteration in the deoxyribose stretching vibration band intensity arising from the DNA backbone (~ 1052 cm^−1^). Low DNA yield has been associated with decreased intensities of specific infrared absorbance bands before^50^.

Dried saliva samples may have great potential for diagnostic purposes. In our study we show that rapid air-drying of saliva supplemented with sucrose on filters can be used to safely store specimens under ambient conditions for later DNA extraction. Furthermore, we show that in situ infrared spectral analysis holds great promise for rapid analysis of biomolecular changes of samples, related to quality assessment and/or rapid disease diagnostics.

## Materials and Methods

### Human saliva collection, and assessments on cell composition and membrane intactness

All methods and procedures were carried out in accordance with guidelines and regulations of NIFE (Lower Saxony Centre for Biomedical Engineering, Implant Research and Development), a jointly shared institution of the Hannover Medical School, the University of Veterinary Medicine Hannover, and the Leibniz University Hannover. Human saliva was obtained from healthy volunteers with informed consent. Use of saliva was carried out after ethical approval according to legal provisions and rules of the Hannover Medical School (Ethics Committee of Hannover Medical School (MHH)). Human saliva samples were collected from healthy volunteers; 20 individuals, two collections each, 13 males and 7 females with ages ranging from 20 − 50 years. The health status of the volunteers was self-assessed. Up to 30 min prior to saliva collection, the volunteers did not consume any foods or drinks. Saliva was collected using two different approaches; namely via spitting in a tube and by applying a cotton swab inside of the cheek. Saliva collected via spitting was mixed with an equal volume (i.e. 100 µL each) of phosphate buffered saline (PBS; 137 mM NaCl, 2.7 mM KCl, 10 mM Na_2_HPO_4_, 1.8 mM KH_2_PO_4_, pH 7.4). Cotton swabs were rubbed against the inside of both cheeks, 6 times each, followed by 1 min immersion in PBS (total volume 100 µL). Saliva collection via the two collection methods was done on the same day with an interval of 2 h between the two collections.

For microscopic evaluations on cell concentration, composition, and membrane intactness; specimens suspended in PBS were mixed [1/2 (v/v)] with 0.5% (w/v) trypan blue staining solution (Serva), and loaded into a hemocytometer (Neubauer-Improved; Karl Hecht). Membrane intact cells exclude trypan blue, whereas damaged cells exhibit intracellular blue staining. In addition to determining numbers of membrane intact/damaged cells, the cell type was inventoried (i.e. buccal cells, leucocytes), and the cell concentration was determined (i.e. in the originally recovered saliva or swab/sample in PBS).

### Drying of saliva, determination of drying kinetics, and storage conditions

The spiting collection method was used to assess stability of dried saliva specimens. A minimum of 5 mL saliva was collected per individual in a 15 mL polypropylene tube (Sarstedt). Samples were vortexed for 10 s, where after 2 mL aliquots were transferred into microtubes for centrifugation (5,000 × g, 5 min). The obtained cell pellet was resuspended in 1 mL PBS. Thereafter, specimens were diluted 1/1 (v/v) with PBS with(out) 20% (w/v) sucrose; finally resulting in saliva samples without protective supplements or with 10% sucrose added. One-mL samples were then added onto polyvinylidene fluoride (PVDF) circular membrane filters (~ 5 cm diameter), which had a 0.22 µm pore size (Merck). Filters with saliva were positioned in a Styrofoam box and dried in the presence of a stream of dry air (< 3% RH). Standard laboratory compressed air was passed through a compressed air dryer (PNEUDRI MiDAS, Parker), which creates dry air with an RH < 3%. The dry air was led through the Styrofoam box with the samples. In addition to drying on filters under a stream of dry air (referred to as ‘fast drying’), alternative drying approaches were tested. These included ‘ordinary drying’ on filters at ambient conditions, and applying a stream of dry air for ‘fast drying of saliva samples on a plastic surface’.

To monitor the drying kinetics of water evaporation for the different drying approaches, a K-type Thermocouple (Fluke) was inserted in the saliva sample; and the temperature was monitored versus the drying time. Water evaporation causes a reduction in sample temperature, which is referred to as evaporative cooling.

Saliva samples, obtained using the spiting collection method, and dried on filters in the presence of a stream of dry air, were stored for up to 90 d at various temperatures (4, 22, 37 °C) as well as for 30 d at different relative humidity (50, 75, 95% RH) at room temperature. In case when varying the storage temperature, the humidity was controlled by sealing the dried specimens in plastic bags (to avoid moisture uptake of the samples). Storage at different relative humidity was done by placing the dried samples in a container using a saturated NaCl solution or water to create a relative humidity of respectively, 75 and 95%, within the container. Storage at 50% RH, was done by exposing the samples to ambient laboratory conditions. Samples were stored in darkness, and six samples were tested per condition.

### Determination of DNA content, of various saliva samples

Total DNA was recovered from saliva samples collected using two different approaches (via spitting and via cotton swab) as well as from saliva dried on filters. Extraction of DNA was realized using the commercially available QIAamp DNA Mini Kit (Qiagen). Different extraction protocols were performed for these different sample types; DNA purification from bodily fluids, DNA purification from buccal swabs, and DNA purification from dried spots, and extractions were done according to the instructions provided by the manufacturer. DNA from specimens was finally eluted from the provided silica columns using 100 μL elution buffer (10 mM TRIS–HCl, 1 mM EDTA, pH 8.0). The DNA content and purity were assessed spectrophotometrically using a Nanodrop 2000 spectrophotometer (Thermo-Fisher) by measuring the absorbance values at 260 nm and 260 versus 280 nm, respectively.

### Fourier transform infrared spectroscopy, and spectral analysis

Infrared spectra of saliva samples dried on filters were collected using a Nicolet iS5 FTIR spectrometer (Thermo-Fisher) equipped with a triglycine sulfate detector and an attenuated total reflection (ATR) accessory, with pressure arm and a diamond/ZnSe crystal. Spectra analysis was done using the accompanying OMNIC Spectra Software (Thermo-Fisher).

Saliva samples dried on filters were analyzed by mounting the samples on the ATR-crystal using the pressure arm. In addition, DNA extracted from filters was analyzed by putting 5 µL recovered DNA solution (10 ng µL^−1^ in elution buffer) on the ATR crystal and allowing it to evaporate over time (air-drying of 5 µL droplets typically takes 15–30 min). Spectra acquisition parameters used were: 4 cm^−1^ resolution, 4 co-added interferograms, and 4000–900 cm^−1^ wavenumber range.

The 1700–1600 and 1400–900 cm^−1^ spectral regions are referred to as the Amide-I and fingerprint region, respectively. These spectral regions were selected and normalized, where after second derivative spectra were calculated with a 21-point smoothing factor using the Savitzky-Golay method. The latter was done to better resolve the absorbance bands representing α-helical and β-sheet structures at ~ 1650 and ~ 1630 cm^−1^, respectively. Differences amongst samples were quantified by calculating the ratio of the intensities of these bands [i.e. I(*v*1630)/I(*v*1650)]. Furthermore, the ratio of the intensities of the absorbance bands representing asymmetric and symmetric PO_2_^−^ stretching vibrations, at respectively 1240 and 1080 cm^−1^, were calculated [i.e. I(*v* 1240)/I(*v* 1080)].

In addition, principal component analysis (PCA) was used to analyze FTIR spectra obtained from different treatment groups. PCA allows assessing differences among spectra, by applying multivariate analysis and reducing the number of variables in a multidimensional dataset^18^. The main goal of PCA is to obtain a small set of principal components (PC) that explain most of the variability in the data sets. Prior to performing PCA, the finger print region (1400 − 900 cm^−1^) was selected, and subjected to a linear base line correction and vector normalization. For vector normalization, spectra are first mean-centered by calculating the average value of the absorbance values for the selected spectral region. This value is then subtracted from the spectrum, where after spectra are scaled such, that the sum squared deviation over the indicated wavelengths equals one:

1 $$a_{m} = \frac{{\mathop \sum \nolimits_{k} a\left( k \right)}}{N\left( k \right)}$$

2 $$a^{\prime } \left( k \right) = a\left( k \right) - a_{m}$$

3 $$a^{\prime\prime}\left( k \right) = \frac{{a^{\prime}\left( k \right)}}{{\sqrt {\mathop \sum \nolimits_{k} (a^{\prime}\left( k \right))^{2} } }}$$

4 $$\mathop \sum \limits_{k} (a^{\prime\prime}\left( k \right))^{2} = 1$$

Here a(k) reflects the spectral intensity at wavenumber k, and N(k) the total number of discrete wavenumbers in the selected spectral region. Covariance-based PCA was performed using MATLAB (Mathworks). Plots were constructed in which PC1 was plotted versus PC2, to visualize differences among treatment-groups.
